# From regulation to real legal cases: physician–patient disputes as a lens on universal health coverage governance in China, 2015–2025

**DOI:** 10.3389/fpubh.2026.1902179

**Published:** 2026-08-12

**Authors:** Hong Pan, Fanlin Meng, Zohar Lederman, Weitong Zhang, Lingqiao Song

**Affiliations:** 1 The Second People’s Hospital, Wuhu Hospital, East China Normal University, Wuhu, China; 2 School of Philosophy and Law and Political Science, Shanghai Normal University, Shanghai, China; 3 Department of Emergency Medicine, LKS School of Medicine, The University of Hong Kong, Hong Kong, China; 4 Centre for Medical Ethics and Law, The University of Hong Kong, Hong Kong, China; 5 Centre of Genomics and Policy, Faculty of Medicine and Health Science, McGill University, Montreal, QC, Canada

**Keywords:** legal analysis, physician-patient dispute, policy recommendations, regulatory framework, universal health coverage

## Abstract

Physician–patient disputes (PPD) have been increasingly recognized as structural indicators of health system performance, yet they remain underexplored within universal health coverage (UHC) frameworks, particularly in the Chinese context. This study employed a mixed-methods approach integrating regulatory review and legal cases analysis, revealing that the regulatory framework for PPD has evolved from fragmented arrangements toward a more integrated civil, criminal, and administrative system, encompassing three stages: ex ante prevention, in-process management, and ex post dispute resolution and sanctioning. We analyzed 4,243 physician–patient disputes cases (2015–2025), comprising 4,086 civil, 56 criminal, and 101 administrative cases. A marked decline was identified in publicly available dispute cases after 2019. This decline was temporally associated with major regulatory reforms and the COVID-19 pandemic, although the descriptive design of this study does not establish strong causation. The underlying structural drivers remain unresolved: 51.3% of civil cases arose from patients' subjective perception of care failure rather than confirmed clinical fault, may point to a systemic deficit in physician–patient communication. Criminal cases were disproportionately associated with critical illness and high financial burden, suggesting that incomplete financial protection may contribute to extreme forms of physician–patient conflict. Over 91.7% of all disputes were concentrated in tertiary hospitals, reflecting potential structural imbalances in resource distribution. This study suggests that PPD may be a form of institutional risk transfer originating from systemic deficiencies within the UHC framework. Legal governance alone may not be sufficient to achieve UHC goals; effective system performance ultimately depends on trust, equity, and accessibility within the health system. Future policy may consider strengthening security measures in high-risk departments, improving financial protection for high-cost conditions, developing communication-support roles, strengthening family doctor services at lower-level institutions, and testing patient engagement mechanisms to support shared decision-making.

## 1 Introduction

Universal health coverage (UHC) has become a central goal of global health systems, aiming to ensure that all individuals have access to needed healthcare services without financial hardship (1). UHC is commonly understood through three interrelated dimensions: access to care, quality of services, and financial protection (2). UHC in China has developed rapidly since the 2009 healthcare reforms, establishing a multi-level insurance system consisting mainly of the Urban Employee Basic Medical Insurance (UEBMI), the Urban and Rural Resident Basic Medical Insurance (URRBMI), and supplementary critical illness insurance schemes (3). By the early 2020s, over 95% of the population was covered by basic medical insurance (4).

However, achieving nominal UHC, measured by insurance enrollment rates and coverage breadth, is distinct from achieving effective UHC, which requires that covered individuals actually experience care as accessible, equitable, financially protected, and trustworthy (5, 6). This study uses PPD as medico-legal signals rather than direct measures of UHC performance. The analytical linkage between PPD and UHC lies in the way different dispute patterns correspond to core dimensions of effective coverage. Disputes concentrated in tertiary hospitals may indicate problems of access and equity, particularly where patients bypass lower-level institutions because of limited trust in primary care. Civil disputes involving alleged malpractice or perceived care failure may reflect concerns about service quality, patient-centered communication, and institutional trust. Criminal cases involving critical illness and high-cost treatment may point to gaps in financial protection, especially when patients and families face severe out-of-pocket burdens. In this sense, PPD provide a practical lens for examining whether formal health coverage translates into accessible, equitable, financially protective, and trusted care in real-world clinical encounters.

In China, physician-patient relationship (PPR) has long been embedded in traditional Chinese medical ethics, where trust, sincerity, and moral responsibility were regarded as essential to healing (7). Classical medical thought associated with Bian Que emphasized that successful treatment depends not only on medical technique but also on the patient's willingness to trust and cooperate with the physician (8). This ethical orientation was further systematized by Sun Simiao, whose writings stressed that physicians should treat patients with compassion, fairness, and sincerity regardless of social status (9). Over time, these traditions contributed to a culturally embedded understanding of medicine as a trust-based moral relationship rather than a purely technical service (10).

This historically grounded ideal has not disappeared in modern China. Rather, it has increasingly been incorporated into contemporary governance discourse. Regulatory instruments such as the *Regulation on the Prevention and Handling of Medical Disputes* and broader policy agendas such as *Healthy China 2030* reflect an institutional effort to rebuild harmonious PPR through communication, procedural fairness, and dispute prevention (11). As one of the most important dimensions of the PPR, PPD provide a direct lens through which tensions within the healthcare system can be examined.

However, existing scholarship has not sufficiently explained how PPD can function as a structural indicator of UHC performance, nor has it adequately translated this insight into constructive governance recommendations. Moreover, limited research has integrated regulatory analysis with real-world medico-legal data to examine how PPD reflect deeper systemic tensions related to access to care, quality of services, financial protection, and institutional trust.

This study examines PPD within the context of China's UHC system, drawing on interdisciplinary collaboration among three legal researchers (Song L., Meng F. Zhang W.) and two frontline healthcare professionals (Pan H., Ledman Z.) across the Greater China Area (Hong Kong and Mainland China). This article addresses the following research question: How China's evolving legal and regulatory frameworks, together with publicly available PPD cases between 2015 and 2025, reveal the gaps between nominal and effective universal health coverage.

## 2 Methods

This study employed a mixed-methods approach, combining regulatory analysis with legal case analysis, to examine issues surrounding physician–patient disputes (PPD) in the context of China's universal health coverage (UHC).

First, two legal researchers (Song L. and Meng F.) conducted a systematic review of national and local laws and regulations from 2002 to 2025 using legal databases, including Alpha and Pkulaw. Keywords including “医患纠纷” (physician–patient disputes), “医疗损害责任纠纷” (medical malpractice disputes), “医患关系” (physician–patient relationship), and “暴力伤医” (violence against healthcare practitioners) were used to identify relevant normative documents. Documents were excluded if they were ineffective, repealed, superseded by subsequent legislation, or lacked direct regulatory relevance to PPD. The final set of 83 laws and regulations (comprising 5 are national laws, 8 are regulations, and 70 are local regulations) was analyzed to examine governance mechanisms related to dispute prevention, liability allocation, mediation procedures, and legal sanctions.

Second, a legal case search concerning PPD between 2015 and 2025 was conducted using the Alpha database and China Judgments Online with the same search terms. Cases were included if they involved disputes between patients and physicians or affiliated medical institutions, allegations that patients' personal or property rights had been infringed upon, or violations of healthcare professionals' personal rights. Given that some judgments involving confidential or personal information are not fully disclosed online, the study supplemented the legal database search with official media reports from the People's Daily, Guangming Daily, and CCTV News concerning violence against medical personnel.

An initial dataset of 5,889 cases was identified. After deduplication, completeness assessment, and relevance screening, 4,243 valid cases were retained, including 4,086 civil cases, 56 criminal cases (30 from media search), and 101 administrative cases (77 from media search).

The legal databases used in this study primarily cover publicly available court judgments and therefore do not capture the full universe of physician–patient disputes in China. Disputes resolved through mediation, private settlement, administrative complaint mechanisms, internal hospital procedures, or unpublished judgments may not be included. Accordingly, the dataset should be understood as a collection of publicly available legal and medico-legal cases rather than a comprehensive record of all PPD incidents during the study period.

Media reports were used only to supplement the identification of PPD incidents that were not fully available in court databases. They were not used for detailed legal coding unless sufficient factual information was available. This approach improves coverage of severe incidents but may introduce reporting bias, since media-reported cases are more likely to involve serious violence, public disorder, or high-profile events.

## 3 Assessment of regulatory framework and implications: three-tiered regulatory framework for physician–patient disputes: from ex ante to ex post

**Figure 1:**
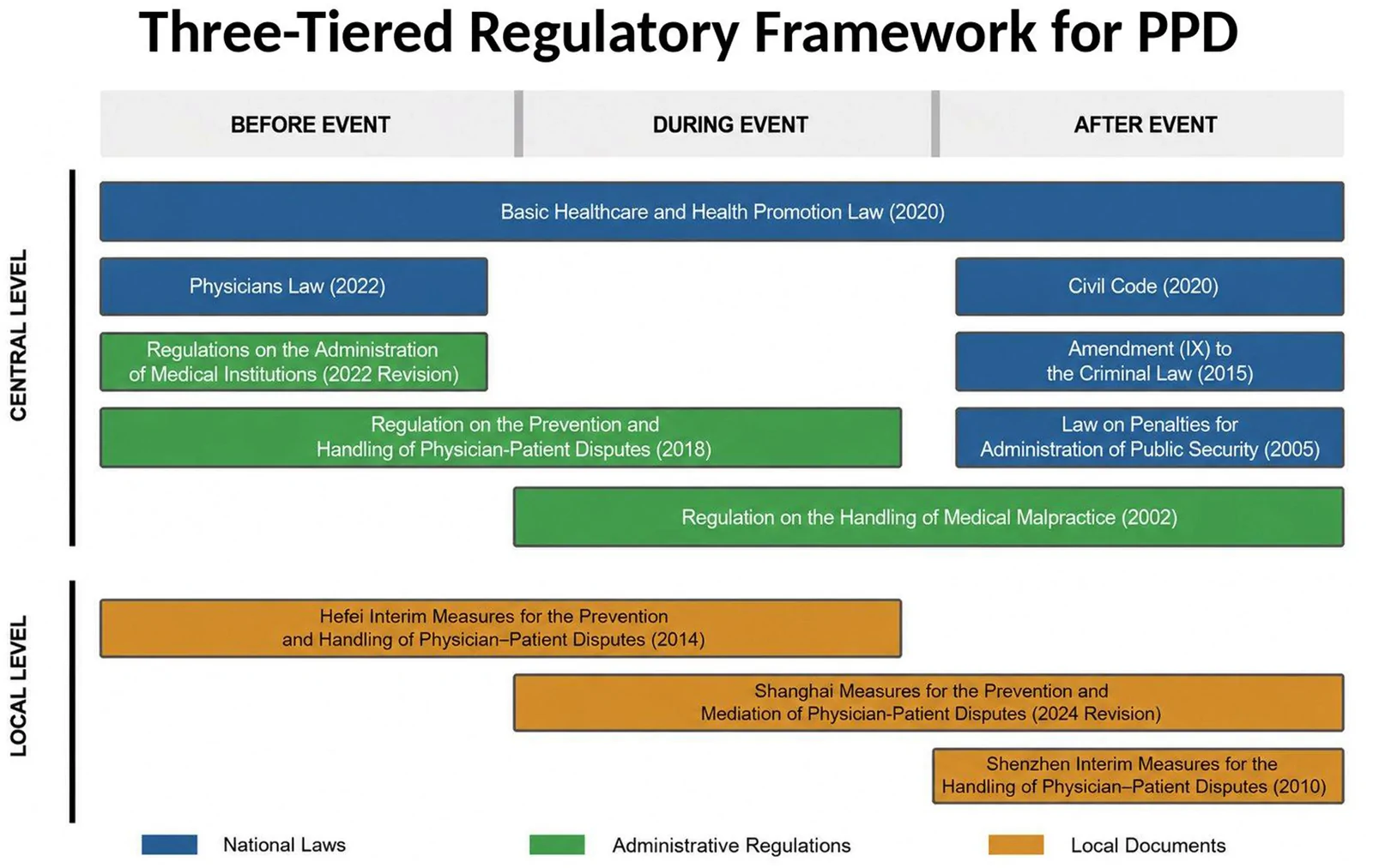
Three-tiered regulatory framework for PPD (local regulations are only selected for demonstration of three-tier legal and regulatory framework).

China has established a three-tier legal and regulatory framework for the prevention and resolution of medical disputes, shifting from a reactive disciplinary approach toward a comprehensive governance model integrating prevention, in-process management, and post-incident resolution. At the preventive level, the *Regulation on the Prevention and Handling of Medical Disputes* (2018) requires medical institutions to establish comprehensive complaint-handling and emergency response mechanisms, strengthen communication with patients, improve medical quality management, and implement risk assessment systems aimed at reducing the occurrence of PPD. The *Regulation* emphasizes early intervention and institutional accountability, reflecting a transition toward proactive dispute prevention rather than solely *post hoc* liability allocation (see Table 1 and Figure 1).

**Table 1:** Laws and regulations governing physician-patient relationships.

Legal hierarchy	Title of document	Year	Governance phase
National Law	Basic Healthcare and Health Promotion Law	2020	Before, During, After
National Law	Civil Code of the People's Republic of China	2020	After (Resolution & Liability)
National Law	Amendment (IX) to the Criminal Law	2015	After (Punitive Sanctioning)
National Law	Law on Penalties for Administration of Public Security	2005	After (Administrative Sanctioning)
National Law	Physicians Law of the People's Republic of China	2022	Before (Professional Regulation)
Administrative Regulation	Regulations on the Administration of Medical Institutions (Latest Revision)	2022	Before (Institutional Compliance)
Administrative Regulation	Regulation on the Handling of Medical Malpractice	2002	During, After
Administrative Regulation	Regulation on the Prevention and Handling of Medical Disputes	2018	Before, During

In addition, the *Regulation on the Handling of Medical Malpractice* (2002) requires healthcare institutions to properly maintain and preserve patients' medical records, standardize clinical procedures, and ensure transparency during medical treatment processes (12). These requirements are intended not only to facilitate evidence collection and liability determination in medical malpractice disputes but also to reduce mistrust and informational asymmetry between physicians and patients, which are commonly identified drivers of PPD.

At the foundational legislative level, the *Basic Healthcare and Health Promotion Law of the People's Republic of China (BHHPL)*, which came into effect in 2020, establishes overarching normative objectives for safeguarding public health, maintaining harmonious physician–patient relationships (PPR), protecting patients' lawful rights and interests, and ensuring the personal safety and professional dignity of healthcare workers. Chapter 9 (“Legal Liability”) of the BHHPL further provides statutory grounds for administrative penalties and legal sanctions against conduct disrupting medical order, including violence against healthcare personnel and disturbances within medical institutions (13).

At the same time, this regulatory framework has evolved from fragmented into a more integrated system combining civil, criminal, and administrative mechanisms in addressing PPD. The *Civil Code (2020)*, constitutes the main mechanism for resolving disputes by defining liability standards for medical harm (14). The *Civil Code* strengthens the protection of patient rights in the context of medical harm liability, particularly through Articles 1218 to 1228 concerning medical malpractice and tort liability. Among these provisions, Article 1219 emphasizes patients' rights to informed consent by requiring medical personnel to disclose medical conditions, treatment risks, and alternative treatment options before conducting surgery or special treatment. In addition, Article 1218 establishes that medical institutions shall bear liability where patients suffer harm due to the fault of medical staff. These provisions collectively reinforce patient autonomy, the right to information, and access to compensation in medical disputes. However, while the *Civil Code* places substantial emphasis on protecting patients' procedural and substantive rights, comparatively less attention is given to the enforcement of patient responsibilities, such as the duty to provide accurate medical information, comply with medical advice, or engage in good-faith communication during treatment (14, 15). This asymmetry may contribute to a perceived imbalance in the allocation of rights and obligations within medical dispute resolution. From the perspective of healthcare providers, an overly patient-centered liability framework may increase defensive medicine practices and intensify concerns regarding professional risk exposure. Consequently, although the legislation aims to enhance patient protection and trust in healthcare, it may also affect perceptions of fairness and reciprocity within the healthcare system if corresponding patient obligations are not equally emphasized (16).

The *Criminal Law* functions as a deterrent but remains insufficient, as PPD are not defined as a distinct category of offense (17). Instead, cases are prosecuted under general provisions such as intentional injury or public order violations. This fragmentation limits the system's capacity to address the specific risks associated with violence in healthcare settings, which can undermine provider safety and, consequently, the availability and quality of care.

Administrative regulations occupy a critical intermediate position in the regulatory framework, bridging the gap between criminal law and civil law. They address conduct that is too serious to be resolved through civil remedies alone yet falls short of the threshold required for criminal prosecution. In the context of PPD, administrative regulations serve a dual function: on one hand, they govern professional sanctions against physicians, including license suspension, revocation, and practice restrictions, for violations of medical standards and professional conduct; on the other hand, they provide mechanisms to address patient misconduct, such as disruptive or violent behavior in medical settings that does not rise to the level of criminal offense. The *Regulation on the Prevention and Handling of Medical Disputes (2018)* has established a full-process governance approach incorporating risk prevention, communication mechanisms, and diversified dispute resolution pathways such as mediation (18).

Beyond the national legal and regulatory framework, local governments have developed corresponding normative instruments based on regional practices, forming a coordinated governance structure between central and local authorities. This study finds that Supreme people's courts, health administrative departments, and local governments in multiple regions have issued specialized policy documents addressing physician–patient relations.

For example, the High People's Court of Zhejiang Province issued the *Opinions on Adjudicating Medical Cases in Accordance with Law and Promoting Harmonious Physician–Patient Relations* (2014), which clarifies adjudication principles and standards from a judicial perspective. The Health Department of Shenzhen released the *Interim Measures for the Handling of PPD* focusing on regulating medical practice from an administrative governance perspective. In addition, the General Office of the Nanjing Municipal People's Government circulated the *Opinions on Promoting Medical Liability Insurance and Advancing People's Mediation in Medical Disputes*, jointly developed by the municipal health and justice authorities, which explores a dispute resolution model combining medical liability insurance with community-based mediation mechanisms. These local normative documents serve as important supplements to the national legal framework and reflect the institutional flexibility and context-specific governance of physician–patient relations through central–local interaction.

Overall, this regulatory framework reflects a shift from reactive punishment toward proactive and process-oriented regulation, with increasingly diversified policy instruments and a growing emphasis on balancing patient rights, provider protection, and social stability. However, persistent structural tensions remain, including the absence of dispute-specific offenses in criminal law and imbalances between patients' rights and their responsibilities in the civil law (19). These findings suggest that the regulatory framework alone may be insufficient to resolve physician–patient disputes. To assess whether the regulatory framework translates into tangible outcomes in practice, Section 2 conducted a legal case analysis of PPD from 2015 to 2025. By examining real cases, Section 2 aims to identify the gaps between the lived dynamics of PPD and the existing legal framework, revealing where formal regulation fails to capture, or adequately address, the underlying tensions within UHC in China.

## 4 Discussion: lessons from legal cases between 2015 to 2025

### 4.1 A turning point in PPD trends around 2019

A total number of 4,243 legal cases were identified related to PPD from 2015 to 2025, comprising 4,086 civil cases (96.3%), 56 criminal cases (1.3%; with 26 from legal databases and 30 from news media), and 101 administrative cases (2.4%; 24 from legal databases and 77 from news media). Civil disputes accounted for most of the sample, indicating that most disputes were resolved through compensation-based legal mechanisms rather than through criminal or administrative channels (see Table 2).

**Table 2:** Legal cases of PPD: a turning point in legal dispute trends around 2019.

Year	Total cases	Civil cases	Criminal cases	Administrative cases
2015	478	463	6	9
2016	626	613	8	5
2017	617	603	6	8
2018	618	602	4	12
2019	526	508	11	7
2020	487	474	5	8
2021	358	348	1	9
2022	197	185	2	10
2023	131	118	5	8
2024	118	102	5	11
2025	87	70	3	14
Total	4,243	4,086	56	101

A key finding of this study is a turning point in publicly available PPD cases around 2019. Prior to 2019, the number of disputes showed a steady increase, which may reflect persistent tensions in physician–patient relations alongside expanding access to legal remedies. After 2019, the number of cases declined markedly and remained at a relatively low level. This decline should be interpreted cautiously. The timing coincides with several overlapping developments, including the implementation of the Regulation on the (43, 44). The COVID-19 pandemic and changes in healthcare utilization, judicial disclosure or reporting practices may also contribute to the decline. Therefore, this study does not claim that legal reform caused the decline in dispute cases. Rather, the observed trend indicates a temporal association between regulatory changes, pandemic-related contextual factors, and reduced publicly available litigation.

The COVID-19 pandemic may have been an important contextual factor between 2019 and 2022, as reduced healthcare utilization, increased social cohesion, and lower public expectations regarding service quality may have contributed to fewer formal disputes (20, 21). However, the continued decline from 2023 to 2025 suggests that other institutional or governance-related factors may also have played a role. Further research using healthcare utilization denominators, mediation records, administrative complaint data, and longitudinal statistical methods would be needed to determine whether the decline reflects a genuine reduction in disputes, a shift in dispute-resolution pathways, or changes in case disclosure.

Table 2 may therefore be read as a descriptive trend in publicly available cases rather than as evidence of a causal effect of regulatory reform. The observed decline in dispute frequency should also not be interpreted as evidence that underlying systemic tensions have been resolved. A healthcare system may produce fewer formal disputes while still facing persistent structural vulnerabilities in financial protection, resource distribution, and communication. This gap between reduced formal conflict and effective systemic improvement is what the following sections seek to examine.

### 4.2 Civil disputes: perception-driven conflicts rather than confirmed medical fault

Under Chinese Civil Code, a finding of medical liability generally requires not only patient injury but also legally established fault by the medical institution or healthcare professional and a causal relationship between the fault and the alleged damage. Accordingly, this study distinguished between “confirmed medical fault” and “patient-perceived breach of duty of care.” Cases were coded as confirmed medical fault where the court, an expert appraisal, or the judgment reasoning expressly established fault and causation. Cases were coded as patient-perceived breach of duty of care where the patient alleged inadequate diagnosis, treatment, explanation, or care, but the judgment did not confirm legally actionable medical fault. Separate categories were used for falsification or tampering of medical records, failure to obtain informed consent, and other or unclear causes. Two legal researchers independently coded the cases according to the above criteria. Disagreements were resolved through discussion with a third legal researcher and, where necessary, consultation with clinical co-authors to ensure that legal categories were interpreted consistently with clinical realities (see Table 3).

**Table 3:** Civil disputes: perception-driven conflicts rather than confirmed medical fault.

Causal attribution	Number	Percentage
Patient-perceived breach of duty of care	2,095	51.3%
Confirmed medical fault	669	16.4%
Falsification of medical records	124	3.0%
Failure to obtain informed consent	11	0.27%
Others (reason undisclosed, unidentified, or miscellaneous, e.g., billing disputes)	1,187	29.1%
Total	4,086	100.0%

As shown in Table 3, civil disputes in this study are predominantly perception-driven rather than grounded in confirmed clinical malpractice.

The largest share, 2,095 cases (51.3%), stems from patients' subjective belief that physicians breach the duty of care, while only 669 cases (16.4%) involve confirmed medical fault. Procedural violations are comparatively rare: 124 cases (3.0%) concern the falsification or tampering of medical records, and 11 cases (0.27%) involve breaches of informed consent requirements.

The relatively high proportion of patient-perceived legal cases, at 51.3%, may indicate a gap between patients' perceptions and legally established medical fault. The prevalence of subjective perception as a conflict driver, however, points to deeper structural and relational deficits. First, a central structural explanation may lie in the asymmetry of medical knowledge between physicians and patients (22). Physicians operate within highly specialized and often opaque knowledge systems, while patients face significant barriers in understanding diagnoses, treatment risks, and clinical uncertainties, barriers that limit meaningful patient participation and weaken the practical implementation of informed consent (23). Second, medical paternalism may be a persistent cultural feature of Chinese clinical encounters, where physicians retain strong decision-making authority with limited space for patient input, potentially leaving patients feeling excluded or disrespected (24). Third, a lack of empathy and relational communication may cause patients to interpret clinically appropriate care as negligent or indifferent (25). Taken together, these factors converge on a possible underlying deficit: the absence of structured, meaningful communication between physicians and patients. Future technologies including Artificial Intelligence (AI), may further complicate communication and knowledge asymmetry in clinical encounters.

Compared with high rate of subjective perception of breach of duty of care, the most “effective” and common communication way: the informed consent was only 11 cases (0.3%) were attributed to failure to obtain informed consent, a figure that appears paradoxically low. This does not necessarily indicate that informed consent is effectively implemented in practice; rather, it may reflect systemic limitations in legal recognition, documentation, and enforcement, meaning that many violations go unrecorded or unlitigated. This analysis suggests that informed consent, as currently practiced, is insufficient as a standalone communication mechanism.

A possible approach would institutionalize dedicated communication roles, professionals responsible not for treatment, but for explanation. Drawing on models such as medical interpreters, patient navigators, and genetic counselors, these roles would translate complex medical records, diagnostic results, and treatment options into accessible language tailored to individual patients. By reducing misunderstanding and bridging the knowledge gap, such mechanisms may improve patient engagement, strengthen trust, and possibly reduce dispute incidence (26–30).

Moreover, patient engagement frameworks, widely developed in North American contexts, most notably Canada, where Manafo et al. (31) documented their application in health research, offer a possible transferable model. While the specific institutional contexts differ, the core principle is applicable to China's PPD challenge: repositioning patients from passive recipients of medical decisions to active participants in their own care. In China's context, where medical paternalism remains prevalent and patient health literacy varies significantly across regions and demographic groups, structured engagement mechanisms could help align patient expectations with clinical realities, directly addressing the perception gap that underlies 51.3% of civil disputes. Adapting such frameworks to China would require accounting for regional disparities in health literacy, the role of family in medical decision-making, and the availability of community-level health infrastructure.

### 4.3 Criminal cases: extreme manifestations of system stress

The criminal case analysis should be interpreted cautiously. Of the 56 criminal cases included in the sample, 26 were court judgments and 30 were derived from news reports. While the court judgments provided detailed factual and judicial information, the news reports generally consisted of preliminary media coverage only. Due to privacy concerns, hospitals and judicial authorities did not disclose further details regarding these reported incidents. Consequently, the criminal case analysis in this study primarily relies on the 26 court judgments. The limited sample renders these findings exploratory and hypothesis-generating, rather than statistically generalizable. They offer insights into severe dispute escalation and potential links between critical illness, financial burden, emotional distress, and systemic healthcare pressures as below (see Table 4).

**Table 4:** Criminal PPD as extreme manifestations of system stress.

Panel A. Case distribution		
Case description	Number	Percentage
Critical or life-threatening illness	12	46.2%
Fractures and other conditions (minor injuries)	3	11.5%
Death of a relative	4	15.4%
The individual became intoxicated and caused a disturbance	3	11.5%
Reasons undefined	4	15.4%
Total	26	100%
Panel B. Legal categorization of criminal cases		
Criminal charge	Number	Percentage
Intentional homicide	10	38.5%
Intentional injury	4	15.4%
Crimes disrupting public order (e.g., picking quarrels and provoking troubles, gathering crowds to disrupt order)	12	46.2%
Total	26	100.0%

First, a substantial proportion of criminal cases are associated with severe or life-threatening medical conditions. Specifically, 12 out of 26 cases (46.2%) involve patients suffering from critical illnesses such as cancer, stroke, myocardial infarction, or serious postoperative complications. These conditions are characterized by high clinical uncertainty, high mortality risk, and often unfavorable outcomes, which may intensify emotional distress and increase the likelihood of conflict escalation. From a universal health coverage (UHC) perspective, this pattern may suggest gaps in financial protection. For example, certain high-cost treatments, particularly some cancer therapies, are not fully covered by basic health insurance, resulting in significant out-of-pocket expenses. According to data from the National Cancer Center and related surveys, the average total cost for cancer patients in China, from diagnosis to the completion of treatment, is approximately 150,000 to 300,000 yuan (32). The reimbursement rate for employee medical insurance at tertiary hospitals is approximately 85%−90%, while the reimbursement rate for urban and rural resident medical insurance is approximately 70%−75% (33). According to the National Bureau of Statistics' 2025 Statistical Bulletin on National Economic and Social Development, China's per capita GDP in 2025 was 99,665 RMB (34). The combination of high fatality rate, clinical uncertainty, emotional burden, and financial stress places patients and their families under extreme pressure, may increase the risk of dispute escalation into criminal incidents (35).

Second, sociocultural factors, particularly alcohol consumption, may be a recurrent contributing factor. Approximately 3 out of 26 cases (11.5%) involve alcohol-related incidents, where patients or their relatives engaged in violent behavior while intoxicated. This suggests that conflict escalation is not solely driven by clinical or institutional factors but may also be shaped by situational and behavioral dynamics.

Third, the legal categorization of these cases may suggest fragmentation in the regulatory response. Among the 26 cases, 10 cases (38.5%) involve intentional homicide, 4 cases (15.4%) involve intentional injury, and 12 cases (46.2%) involve crimes disrupting public order, such as provoking disturbances or assembling crowds (17). The absence of a distinct criminal category addressing violence against healthcare providers may limit both the visibility of such incidents and the development of targeted legal responses.

Although criminal cases represent a small proportion of total disputes, their severity has disproportionate implications for public trust and health system performance. From a UHC perspective, these cases function as a “stress test” of the healthcare system, revealing how gaps in financial protection, communication, and governance can interact with high-risk clinical scenarios to produce extreme conflict.

From a policy perspective, targeted interventions may be suggested for high-risk settings, particularly in departments such as emergency medicine and among patients with critical illnesses. These interventions may operate across several complementary dimensions. First, protection mechanisms for healthcare providers require strengthening, including improved physical security infrastructure, clearer legal deterrents against violence, and institutional protocols for de-escalation, particularly in high-pressure clinical environments where alcohol-related incidents and critical illness cases represent disproportionate risk.

Second, a two-pronged educational approach may be needed, encompassing both public education and professional training. Public education efforts aimed at fostering a more accurate and positive understanding of healthcare professionals may help rebuild institutional trust and temper unrealistic expectations. At the professional level, targeted training for physicians may enhance empathy and communication skills, particularly when caring for patients with untreatable or terminal conditions, and their families, who frequently face compounding emotional, physical, and financial burdens. Such efforts represent an institutional translation of UHC principles into patient-centered care in practice.

Third, integrating commercial insurance as a supplementary layer to basic UHC schemes may help alleviate the financial burden on patients and families, reducing the economic grievances that frequently escalate into formal disputes. More broadly, better implementation of UHC, particularly in terms of coverage depth and resource allocation, is a fundamental prerequisite for creating a more sustainable and supportive working environment for physicians, addressing the structural conditions that contribute to burnout, understaffing, and the erosion of PPR.

### 4.4 Administrative cases: an intermediate layer of conflict governance

Administrative cases (*n* = 101, accounting for 2.39% of the total 4,243 cases). As with criminal cases, since news reports on administrative penalties did not disclose the grounds for the cases or the penalties imposed, this study excluded cases reported in the news and analyzed only the judicial cases available on the Judgment Document Network (*n* = 24). Administrative regulations represent an intermediate layer of governance, occupying a distinct regulatory space, addressing professional misconduct by healthcare providers and disruptive conduct by patients that falls outside the scope of private civil disputes yet does not rise to the level of criminal liability (36) (see Table 5).

**Table 5:** Administrative cases: an intermediate layer of conflict governance.

Governance target	Typical conduct or regulatory issue	Number of cases	Percentage
Healthcare providers or medical institutions	Unauthorized practice, failure to fulfill statutory duties, or irregularities in medical record management	8	33.3%
Patients or relatives	Verbal abuse, physical confrontation, refusal to comply with procedures, property damage, or disruption of medical order	7	29.2%
Other	Cause of dispute unspecified	9	37.5%
Total	Administrative disputes involving either provider-side noncompliance or patient-side disruptive behavior	24	100.0%

Cases involving healthcare providers or institutions constitute a minority of administrative disputes, accounting for approximately 8 cases (33.3%). These cases typically involve professional regulatory violations imposed on physicians or medical institutions, such as unauthorized medical practice, failure to fulfill statutory duties, or irregularities in medical record management, including concealment, tampering, or incomplete documentation. For example, in *Huang Hongying v. People's Government of Guangxi Zhuang Autonomous Region*, the court found that the physicians had performed surgical procedures beyond their licensed scope, and administrative penalties were imposed pursuant to Article 37 of the *Law of the People's Republic of China on the Practice of Medicine* (now revised to the Physician Law).

In contrast, the administrative cases involve sanctions imposed on patients or their relatives, accounting for approximately 7 cases (29.2%). These cases arise when patients or their relatives, dissatisfied with medical outcomes, engage in disruptive behaviors such as verbal abuse, physical confrontation, refusal to comply with hospital procedures, or disruption of medical operations. For example, in *Liu Xinghong v. Yongchuan District Public Security Bureau of Chongqing Municipality* (first-instance administrative judgment), the defendant and associates attended the hospital to dispute a medical outcome and, refusing to heed staff warnings, proceeded to assault and verbally abuse medical personnel, damage office equipment, and seriously disrupt the hospital's normal operations. Administrative penalties were subsequently imposed for their conduct.

This distribution highlights an important governance dynamic: while civil litigation primarily addresses compensation (4,086 cases) and criminal law targets severe violence (56 cases), administrative mechanisms serve as an intermediate regulatory tool for maintaining order and managing conflicts that fall below the criminal threshold. In particular, administrative sanctions play a preventive role by intervening before disputes escalate into criminal incidents.

From a universal health coverage (UHC) perspective, this dual structure reflects two interconnected challenges. On the one hand, provider-related administrative cases point to deficiencies in service quality, procedural compliance, and institutional accountability, which may undermine patient trust and equity in care. On the other hand, patient-related administrative cases reflect unmet expectations, communication failures, and systemic pressures that contribute to behavioral escalation and disrupt healthcare delivery.

Although the sample is too small to draw definitive conclusions (*n* = 24), the near-equal distribution of provider-side (8 cases) and patient-side (7 cases) administrative sanctions is noteworthy. It suggests that administrative enforcement does not systematically favor either party, and may function as a genuinely intermediate regulatory tool, one that holds both healthcare providers and patients accountable within a single governance framework. Larger-scale administrative case analysis would be needed to confirm this pattern.

### 4.5 Resource disparity: inequality under universal health coverage

Among the 4,243 cases in this dataset for which hospital level was identifiable, 3,891 (91.7%) involved tertiary-level institutions. Hospital level was determined based on the hospital name recorded in the court judgment or media report, cross-referenced against the national hospital classification registry. These departments are characterized by high patient volumes, clinical urgency, and elevated uncertainty, which increase both operational pressure and the likelihood of conflict. Of note, the analysis of hospital-level distribution is based on raw case counts rather than utilization-adjusted dispute rates. Because hospital-level denominators, including outpatient visits, inpatient admissions, emergency volume, and case-mix complexity, were not available, this study cannot determine whether tertiary hospitals have a higher dispute rate per visit or admission.

This distribution may be related to a “resource siphoning” effect, whereby higher-level hospitals in developed regions attract large numbers of patients from other areas. Two mechanisms may be relevant to this effect (37). First, higher patient volumes increase the absolute number of disputes due to a larger service base. Second, patients with more severe and complex conditions tend to concentrate in tertiary hospitals, where clinical uncertainty, emotional stress, and financial burden are significantly higher, further increasing the risk of disputes.

From a UHC perspective, this pattern suggests a structural imbalance between demand and supply. While UHC expansion has increased healthcare utilization, through expanded insurance coverage, the supply of high-quality medical resources, such as experienced physicians and hospital beds in tertiary institutions, has not increased proportionally (38). As a result, tertiary hospitals face excessive patient loads, prolonged waiting times, and reduced consultation time per patient, which may negatively affect service quality and exacerbate physician–patient tensions (39).

At the same time, lower-level healthcare institutions remain underutilized, may leading to a “hollowing out” of primary care (40). This imbalance may reflect the incomplete implementation of a hierarchical medical system and weak gatekeeping mechanisms. Limited trust in primary care providers further may reinforce patient preferences for tertiary hospitals, creating a self-reinforcing cycle of overconcentration and system inefficiency (41).

To address these challenges, policy efforts may focus on strengthening primary care capacity and promoting the effective use of lower-level healthcare resources. In particular, the development of a family doctor system may improve continuity of care, enhance communication, and rebuild trust at the community level (42). From a UHC perspective, such reforms may improve equity, optimizing resource allocation, and reducing systemic pressures that contribute to PPR.

## 5 Policy implications and conclusion: from regulation to trust-based governance under universal health coverage

This study suggests that physician–patient disputes (PPD) may be more than legal conflicts; they may also serve as medico-legal indicators of structural tensions within China's universal health coverage (UHC) system. Although China has developed a regulatory framework encompassing ex ante prevention, in-process management, and ex post dispute resolution, the legal case analysis suggests that underlying systemic pressures may remain unresolved.

From a UHC perspective, these findings suggest that expanding coverage alone may be insufficient. The core challenge may lie not only in nominal access to healthcare, but also in the quality, equity, and effectiveness of that access, as well as whether patients experience care as trustworthy, fair, and responsive to their needs.

The case-level analysis points to three areas that may warrant further policy attention. First, communication failure and knowledge asymmetry may contribute to perception-driven disputes. The finding that 51.3% of civil disputes involved patients' subjective perception of care failure suggests a gap between patients' experience of care and legally established medical faults. Medical paternalism and insufficient empathy may further compound this dynamic. Potential institutional responses may include dedicated communication-support roles, with professionals responsible for explaining diagnoses, treatment risks, and medical records in accessible terms, analogous to genetic counselors in precision medicine contexts. Structured patient engagement frameworks may also help align expectations, strengthen trust, and reduce perception gaps.

Second, the concentration of disputes in tertiary hospitals may be consistent with inequitable distribution of medical resources, high patient volumes, and complex case mixes. Strengthening primary care and developing family doctor systems may help build long-term physician–patient relationships and reduce pressure on tertiary hospitals. High-pressure departments, including emergency medicine, oncology, and intensive care, may also benefit from targeted protection and communication-support measures.

Third, the cases may point to possible gaps in financial protection. High-cost treatments, particularly for cancer and other critical conditions, may impose substantial financial burdens on patients and families, which could contribute to dispute escalation. Supplementary commercial insurance, alongside improved public coverage, may help reduce out-of-pocket burdens.

Overall, this study suggests that achieving effective UHC may require a shift from a system focused primarily on legal regulation and coverage expansion toward one that also emphasizes communication, trust, equity, and integrated governance. PPD may therefore be understood as a useful indicator for identifying where nominal coverage has not yet been translated into effective, trusted, and patient-centered care.

## 6 Limitations and future direction

This study has several limitations. First, due to confidentiality requirements or judicial disclosure policies, some cases may not have been made public, which could result in incomplete coverage of the overall dispute landscape.

Second, although a legal cases search was conducted using the Alpha, Pkulaw China, and Judgments Online databases during the study period (2015–2025), the coverage may not be exhaustive. Third, because media reports on cases often lack complete case details, the qualitative content analysis was limited to cases for which court judgments were available; consequently, the relatively small number of criminal cases (*n* = 26) and administrative cases (*n* = 24) limits the generalizability of the findings in these categories.

Future research that covers a longer time span, draws from a broader range of data sources, and incorporates more disputes resolved through mediation or informal means will help validate and expand upon these findings. Future research may also examine whether emerging technologies, including AI-assisted diagnostic tools, online medical information platforms, and precision medicine, reshape physician–patient communication and dispute formation. These technologies may reduce knowledge asymmetry for some patients, but they may also generate new misunderstandings where AI-generated information conflicts with physicians' clinical assessments.

Despite these limitations, the dataset provides a systematic and policy-relevant analysis of PPD in China, offering empirical evidence regarding the governance challenges faced in achieving universal health coverage within a rapidly evolving healthcare environment.
